# Common Variable Immunodeficiency and Circulating T_FH_

**DOI:** 10.1155/2016/4951587

**Published:** 2016-03-16

**Authors:** Ana Coraglia, Nora Galassi, Diego S. Fernández Romero, M. Cecilia Juri, Marta Felippo, Alejandro Malbrán, María M. E. de Bracco

**Affiliations:** ^1^Instituto de Investigaciones Hematológicas (IIHEMA), Academia Nacional de Medicina, C1425ASU Ciudad Autónoma de Buenos Aires, Argentina; ^2^IMEX-CONICET, Academia Nacional de Medicina, C1425ASU Ciudad Autónoma de Buenos Aires, Argentina; ^3^Unidad de Alergia, Asma e Inmunología Clínica, C1035AAT Ciudad Autónoma de Buenos Aires, Argentina

## Abstract

CD4+ T follicular helper cells (T_FH_) were assessed in adult patients with common variable immune deficiency (CVID) classified according to the presence of granulomatous disease (GD), autoimmunity (AI), or both GD and AI (Group I) or the absence of AI and GD (Group II). T_FH_ lymphocytes were characterized by expression of CXCR5 and PD-1. T_FH_ were higher (in both absolute number and percentage) in Group I than in Group II CVID patients and normal controls (N). Within CXCR5+CD4+ T cells, the percentage of PD-1 (+) was higher and that of CCR7 (+) was lower in Group I than in Group II and N. The percentages of Treg and T_FH_ reg were similar in both CVID groups and in N. T_FH_ responded to stimulation increasing the expression of the costimulatory molecules CD40L and ICOS as did N. After submitogenic PHA+IL-2 stimulation, intracellular expression of T_FH_ cytokines (IL-10, IL-21) was higher than N in Group I, and IL-4 was higher than N in Group II. These results suggest that T_FH_ are functional in CVID and highlight the association of increased circulating T_FH_ with AI and GD manifestations.

## 1. Introduction

Common variable immunodeficiency (CVID) comprises a heterogeneous group of diseases characterized by abnormal antibody production [1]. It is the most commonly diagnosed primary immunodeficiency, with an incidence of 1/10 000 to 1/50 000. It affects both sexes equally and the clinical manifestations may begin at any age [2–4]. Over 90% of the patients present with recurrent acute and sometimes chronic bacterial infections mainly of the respiratory and gastrointestinal tracts. Another feature of these patients is their major susceptibility for autoimmunity (AI) manifestations, granulomatous diseases (GD), and cancer [5–10]. Diagnosis of CVID is based on a significant decrease of IgG (at least two standard deviations below the mean for age) associated with a decrease of IgA and/or IgM isotypes, in patients older than 4 years of age and absence of isohemagglutinins and/or poor response to polysaccharide vaccines, with other defined causes of hypogammaglobulinemia excluded [2–4, 11].

In most cases the etiology of CVID is not known. In a small percentage of patients, most families stranded, a few genetic defects have been described. These include mutations in genes coding for the inducible costimulator (ICOS) [12], CD19 [13, 14], CD20 [15], CD8 [16], and the B cell-activating factor receptor (BAFF-R) [17]. Eight to 10% of the patients carry mutations in the gene for the B cell receptor transmembrane activator and calcium-modulating cyclophilin ligand interactor (TACI). Similar mutations are found in unaffected relatives and normal controls, suggesting association but not causality [10, 18–23].

The process of acquiring an adequate antibody immune response is complex and involves specialized groups of T lymphocytes that interact with B lymphocytes in a tightly regulated way in the secondary lymphoid organs. This ultimately results in the generation of different immunoglobulin isotypes, memory effector, and regulatory cells. Failure at any of the stages of the process may lead to humoral immunodeficiency.

T follicular helper cells (T_FH_) have a central role in the generation of the germinal center reaction (GC) which is necessary for the correct maturation of the humoral immune response. Their absence or functional impairment generates defects in the assembly of B cell memory that lead to hypogammaglobulinemia. Most studies concerning the role of this T helper subset have been done in animal models, but in humans there is evidence that a group of CD4+ T lymphocytes characterized by the presence of the chemokine receptor CXCR5 reflects T_FH_ present at the lymph nodes [24–26]. Programmed-death 1 (PD-1) is highly expressed in germinal center T_FH_ and its expression is induced after activation in CD4 and CD8 cells [27–29]. The inhibitory role of the PD-1/PD-ligand (PDL-1 or PDL-2) axis has been defined in relation to the T cell mediated response to antigens, but its role in the regulation of the B cell responses is less clear. While some studies report attenuated antibody responses where the PD-1/PDL-1 and PDL-2 interactions are prevented [30], others observed heightened immune responses in coincidence with increased T_FH_ numbers [31].

Circulating T_FH_ are phenotypically and functionally heterogeneous. A subgroup of T_FH_ with central memory/resting profile expressing chemokine receptor 7 (CCR7) predominates in normal individuals, while in patients with autoimmune conditions, T_FH_ with high expression of programmed-death 1 (PD-1) and low CCR7 are more abundant [32]. In addition, a subgroup of T_FH_ expressing both CXCR5 and FoxP3 (T_FH_ reg) that may suppress T_FH_ activity has been described [33]. Antibody deficiency and immune dysregulation in CVID could be related to absence or inefficient function of T_FH_, to increased T_FH_ reg action leading to downregulation of T/B cell cooperation for the synthesis of antibodies, or to interference with T_FH_ action due to other regulatory lymphocytes as CD8+ regulatory cells [34]. Based on these observations we decided to study the characteristics and function of T_FH_ in peripheral blood of adult CVID patients divided into two clinical phenotypes according to the presence or absence of autoimmunity (AI) and/or granulomatous disease (GD).

## 2. Material and Methods

### 2.1. Subjects

We reviewed the clinical and epidemiological data of our cohort of twenty-one adult patients with CVID. The diagnosis of CVID was made according to standard criteria [2, 3], and all subjects were on monthly immunoglobulin replacement therapy. Based on patients' clinical records, we analyzed the age at onset of infectious symptoms, age at diagnosis, age at the time of the study, length of follow-up from CVID diagnosis to time of the study, and clinical manifestations. Patients were divided into two categories: Group I (*n* = 8), patients with AI, GD, or both, and Group II (*n* = 13), patients without these clinical manifestations.

Blood samples were drawn before the infusion of immunoglobulin. All studies were performed blinded to the clinical manifestations of the immunodeficiency. Controls (*n* = 19) were age related normal blood donors (N). This study was approved by the Ethics Committee of Academia Nacional de Medicina. All donors gave informed consent.

### 2.2. PBMC Isolation and Culture

Peripheral blood samples from all subjects were collected on heparin. PBMC were obtained by Ficoll-Hypaque (FH) centrifugation and suspended to 1 × 10^6^/mL in RPMI tissue culture medium containing 10% fetal calf serum (GIBCO, Grand Island, USA), streptomycin, and penicillin (RPMI-FCS). PBMC cultures were carried out in round bottom 5 mL polystyrene tubes (Falcon) containing 2 × 10^6^ PBMC that were suspended in 2 mL RPMI-FCS [35].

### 2.3. Cell Surface Phenotype of Mononuclear Cells from CVID Patients and N Controls

For assay of the different types of lymphocytes, flow cytometry techniques with a three-color assay were used. Aliquots of 100 *μ*L heparinized peripheral blood were incubated with monoclonal antibodies and then were lysed using FACS Lysing solution (Becton Dickinson, San Jose, CA, USA) following the manufacturer's instructions. Analysis of surface markers was performed on a FACScan cytometer (Becton Dickinson, San Jose, CA, USA) and analyzed with FCS Express software. Lymphocytes were selected according to size (FSC) and side (SSC) scatter profiles. PBMC were also processed both before and after cell culture. Fluorescein isothiocyanate (FITC), phycoerythrin (PE), Alexa 488, or peridinin chlorophyll protein (PerCP) anti-human labelled monoclonal antibodies were used according to the manufacturers' instructions. Anti-CD4 (clone RPA-T4), CD25 (clone M-A251), CD45RO (clone UCHL1), CXC-chemokine receptors, 5 (CXCR5) (CD185, clone RF8B2) and 7 (CCR7, Clone 3D12), CD19 (clone HIB19), CD27 (clone M-T271), inducible T cell costimulator (ICOS) (CD278, clone DX29), CD40 ligand (CD40L) (CD154, clone TRAP1), and programmed-death 1 (PD-1) (CD279, clone MIH4) antibodies were purchased from BD Pharmingen, BD, and eBioscience (San Diego, CA, USA). Absolute numbers of the different lymphocytes were calculated taking into account the white cell count and the percentage of lymphocytes in May-Grünwald-Giemsa stained blood smears.

The phenotype of viable PBMC from CVID and N donors was analyzed before and after 2 or 7 days of PHA+IL-2 stimulated culture (performed as detailed below for the assay of cytokines). Lymphocyte viability was calculated taking into account the proportion of live lymphocytes in the SSC/FSC dot plots. Appropriate isotype controls were used to define the positive populations.

### 2.4. FoxP3+CD25+ T Cells (Treg) and FoxP3+CXCR5+ T Cells (T_FH_ reg)

For Treg and T_FH_ reg determination, PBMC were stained with PerCP anti-CD4 and FITC anti-CD25 or PerCP anti-CD4 and Alexa 488 anti-CXCR5. Then they were fixed and permeabilized with Fix and Perm (Caltag Laboratories, Burlingame, California, USA) according to the manufacturer's instructions. After permeabilization they were reacted with PE anti-FoxP3 (clone PCH101, eBioScience, San Diego, CA, USA).

### 2.5. Costimulatory Molecules (CD40L and ICOS) and Intracellular Il-10, IL-21, and IL-4 in CVID and N

PBMC (10^6^/mL) were stimulated with  2.5 *μ*g/mL phytohemagglutinin (PHA-P, Sigma, L1668, St. Louis, MO, USA) and 5 UI/mL interleukin-2 (IL-2, Peprotech, New Jersey, USA) and cultured at 37°C in the CO_2_ incubator for 2 days for costimulatory molecules and for 7 days for intracellular cytokines. The time and concentration of PHA and IL-2 were selected on the basis of previous studies [36]. IL-2 was replenished after 5 days of culture. Controls consisted of PBMC cultures performed in the absence of PHA and IL-2. For costimulatory molecules, PBMC were stained with PerCP anti-CD4, Alexa 488 anti-CXCR5, and either PE anti-CD40L or PE anti-ICOS. For intracellular cytokines, twelve hours before harvesting the cell pellets, these were resuspended in RPMI-FCS (10^6^/mL) and 10 *μ*L of 2 *μ*M monensin solution (BD Pharmingen, San Diego, CA, USA) was added to the cell suspensions. After washing, the cells were stained with CD4, CXCR5 monoclonal antibodies for 20 minutes at room temperature and then fixed and permeabilized with the Fix and Perm (Caltag Laboratories, Burlingame, California, USA). Cells were finally stained with anti-IL-10 (clone JES3-9D7), anti-IL-21 (clone 3A3-N2.1), or anti-IL-4 (clone 8D4-8) antibodies (BD Pharmingen, San Diego, CA, USA) and the appropriate isotype controls for 45 minutes at 4°C, washed again, and the percentage of intracellular IL-10+, IL-21+, or IL-4+ CD4+ lymphocytes as well as that of CD4 lymphocytes that coexpressed CXCR5 and IL-10+, IL-21+, or IL-4+ was determined.

### 2.6. Statistical Analysis

Graphical presentation and statistical analysis of the data were performed using GraphPad Prism 4.0 (GraphPad Software, San Diego, CA, USA). Comparisons between groups were analyzed by a one-way ANOVA and a nonparametric Mann-Whitney test. Correlations between samples were calculated using the linear regression model. *P* values of less than 0.05 were considered significant.

## 3. Results

### 3.1. CVID Patients

We analyzed data on 11 male and 10 female CVID patients; the mean age of patients at the time of the study was 49.9 ± 15.8 years (range 21 to 73), median 38.5. The age at onset of disease was available for 15 patients, one patient did not have infections, and mean age was 19.4 ± 15.2 years (range 1 to 50), median 19. Mean age at diagnosis was 35.4 ± 18.2 years (range 13 to 70), median was 31.5 years, and mean follow-up duration was 10.6 ± 7.8 years (range 1 to 24), median 10.5. All patients were on gammaglobulin replacement therapy since diagnosis.

Patients were divided into two categories, Group I, eight patients, and Group II, 13. The clinical and epidemiological data of Group I patients are shown in Table 1. Patients 4 and 5 had AI disease manifestations 13 and 33 years, respectively, before the onset of infectious symptoms. With the exception of patient 1 that was studied under steroid and infliximab treatment [37], none of the patients in Group I received systemic steroids, standard immunosuppressive therapies, or biologic agents at the time of the study.

### 3.2. Absolute Values of T and B Lymphocytes in CVID and N

**Figure 1:**
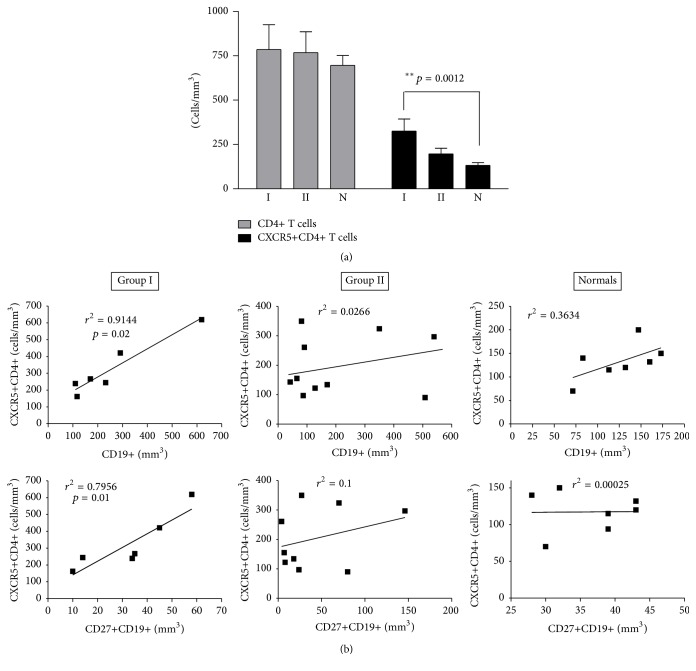
T_FH_ and B lymphocyte absolute values in CVID and N. (a) Absolute lymphocyte values (cells/mm^3^) were calculated in CVID Group I, *n* = 8 (I), and Group II, *n* = 13 (II), patients and control donors (N), *n* = 19. Peripheral blood was stained with monoclonal antibodies and absolute values of viable CD4+ T lymphocytes and CD4+ T cells coexpressing CXCR5 (T_FH_) were calculated after analysis on a FACScan Becton Dickinson flow cytometer. (b) A comparison between peripheral blood viable B lymphocytes (CD19+ cells), memory B lymphocytes (CD27+CD19+ cells), and T_FH_ values (CXCR5+CD4+ cells) was done in CVID Group I, Group II, and N controls. Lineal regression analysis was consigned in the graphs. Only in Group I was the correlation statistically significant.

The total number of lymphocytes, CD4+ T cells, and T_FH_ cells (CXCR5+CD4+) was measured in peripheral blood from CVID patients, right before iv immunoglobulin replacement. As shown in Figure 1, the number of CD4+ lymphocytes was not significantly different in both CVID groups compared to N, but T_FH_ cells were elevated, especially in Group I patients (Figure 1(a)).

In Group I patients the absolute number of CD19+ cells correlated with that of T_FH_ cells suggesting a link in the regulation of these two cell populations (Figure 1(b)). There was no significant correlation between CD19+ cells and T_FH_ in Group II and N. A similar analysis was performed between the absolute number of CD27+CD19+ and those of T_FH_ cells. We found significant correlation only in Group I of patients.

In 2/8 Group I patients and in 6/13 of Group II patients, the percentage of CD19+ B lymphocytes (1.6 to 4.71%) was below normal (N% CD19+, mean + SD: 9.93 + 3.21, *n* = 15).

### 3.3. Follicular T Lymphocytes (T_FH_), T_FH_ Subpopulations, Treg, and T_FH_ reg in CVID and N

**Figure 2:**
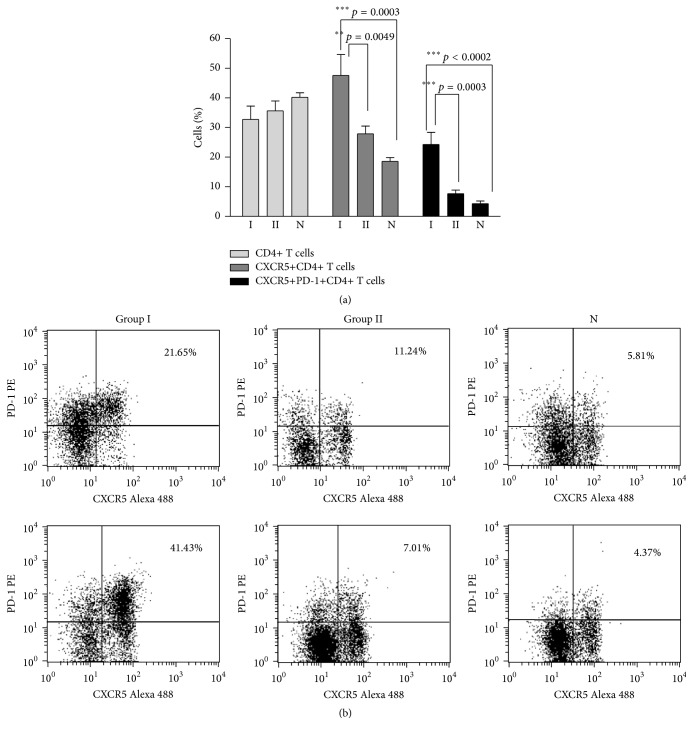
Percentage of T_FH_ (CXCR5+CD4+ T cells) and of T_FH_ coexpressing PD-1 in CVID and N. (a) The percentage of CD4+ T cells and CD4+ T coexpressing CXCR5 or CXCR5+PD-1+ was assayed for CVID patient Groups I (*n* = 7) and II (*n* = 10) and N (*n* = 12). Statistical differences are consigned in the graph. (b) An example of cytometry analysis of two different Group I and Group II CVID patients and two different N controls.

Because CD4 lymphocytes provide essential help for the maturation of B cell memory, we further analyzed the expression of markers associated with follicular helper T cells (CXCR5 and PD-1) in CD4 lymphocytes. The results shown in Figure 2 demonstrate that the percentage of CXCR5+CD4+ T cells was higher in Group I CVID patients when compared to those of Group II and N. Regarding Group II CVID patients, there were no significant differences when compared to N. Concerning coexpression of PD-1 and CXCR5, the highest values were observed in CVID patients with a GD and/or AI disease phenotype (Group I).

In some patients the studies could be repeated after 12–24 months and the CXCR5+PD-1+ percentages in the CD4 region remained high (patient #CM initial value: 23.28%; after 24 months: 25.91%; after 26 months: 25.32%; patient #PV initial value: 15.5%; after 12 months: 18.39%; after 14 months: 16.38%).

**Figure 3:**
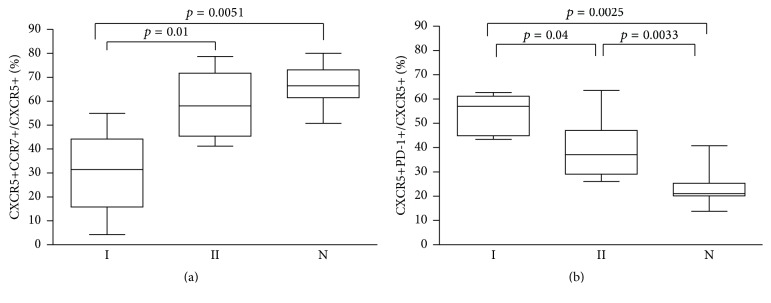
CCR7 and PD-1 expression in T_FH_ of CVID patients. The percentage of CCR7+ cells within the CXCR5+CD4+ T lymphocyte region was calculated for CVID Group I and Group II patients and N (% CXCR5+CCR7+/CXCR5+) (a). Likewise, the percentage of PD-1+ cells in the CXCR5+CD4+ T lymphocyte region was determined (CXCR5+PD-1+/CXCR5+) (b). Statistical differences are consigned in the graph.

Because low CCR7 and high PD-1 expression have been observed in circulating T_FH_ from patients with autoimmune conditions, we examined the T_FH_ subsets considering the level of expression of these molecules on the surface of CXCR5+CD4+ T cells from CVID patients and N controls. The results shown in Figure 3 demonstrate that high PD-1 and low CCR7 expression were the rule in Group I compared to Group II and N. PD-1 was also higher than N in Group II but CCR7 values did not differ from those of N.

The percentages of Treg (CD25+ Foxp3+/CD4) and T_FH_ reg (CXCR5+ Foxp3+/CD4) within the CD4+ T lymphocyte region were similar in both CVID groups and in N (mean ± SD, Group I, Treg: 4.42 ± 1.61%, T_FH_ reg: 3.54 ± 1.38%; Group II, Treg: 4.88 ± 4.61%, T_FH_ reg: 3.21 ± 2.87%; N, Treg: 4.00 ± 2.16%, T_FH_ reg: 2.30 ± 1.83%).

### 3.4. Upregulation of Costimulatory Molecules CD40L and ICOS in CVID and N

Defects in the expression of costimulatory molecules on CD4 lymphocytes could underlie the lack of an adequate humoral response. Therefore we examined CD40L and ICOS expression on CD4 T lymphocytes and CXCR5+CD4+ T lymphocytes after 2 days of culture either nonstimulated (medium) or with PHA+IL-2 stimulation (PHA+IL-2). After T cell stimulation, expression of both CD40L and ICOS could be induced in CD4 cells and in CXCR5+CD4+ T lymphocytes from CVID patients to a similar extent to N (Table 2).

### 3.5. IL-10, IL-21, and IL-4 Induction by T Cell Stimuli in CVID and N

**Figure 4:**
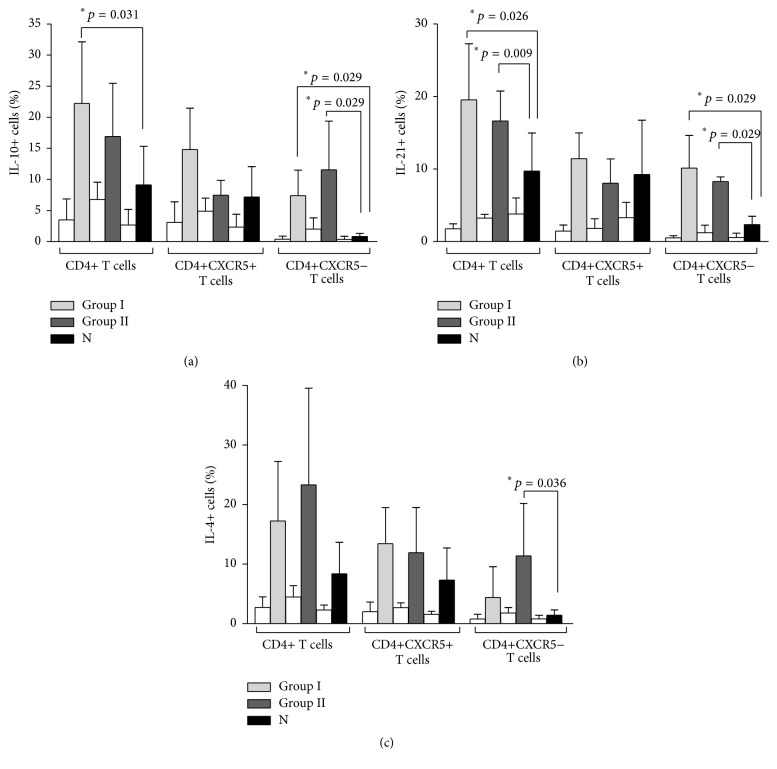
Intracellular cytokines after PHA+IL-2 stimulation of PBMC in CVID and N. The percentage of intracellular cytokines ((a) IL-10, (b) IL-21, and (c) IL-4) in permeabilized CD4+ T cells (CXCR5+ and CXCR5−) was determined after 7 days of PHA+IL-2 stimulation in CVID Group I (*n* = 4), Group II (*n* = 4), and N (*n* = 6). Statistical differences are consigned in the graph. White bars represent unstimulated controls.

Although other subtypes of T helper lymphocytes can synthesize these cytokines, human T_FH_ produce abundant IL-21 and IL-10 upon stimulation. IL-4 is also a CD4-derived cytokine involved in the process of antibody generation and it can be produced by Th2 CD4 lymphocytes and by the Th2 subset of T_FH_ [24]. In order to assess the ability of CD4 T cells to produce these cytokines, PBMC were stimulated with a submitogenic dose of PHA and IL-2. The percentage of CD4 T cells with intracellular IL-10, IL-21, or IL-4 was recorded. The percentage of IL-10+ and IL-21+ CD4+ T cells was higher than N in CVID Group I and Group II after PHA stimulation while IL-4+ CD4+ T cells were not different to N in both CVID groups. When considering cytokine (IL-10, IL-21, and IL-4) expression by CD4+CXCR5+ T cells (T_FH_), it was similar in both groups of CVID to that of N. However, when cytokine expression (IL-10 and IL-21) was analyzed in CD4+ T cells that did not express CXCR5, it was significantly higher (*p* < 0.029) in both CVID groups than in N (Figures 4(a) and 4(b)). IL-4 expression was higher than N (*p* = 0.036) in CXCR5− CD4+ T cells (Figure 4(c)) in Group II patients.

## 4. Discussion

It is difficult to determine the immunopathogenic importance and/or predictive value of different laboratory findings in relation to clinical manifestations in adult CVID. Attempts to correlate clinical observations with laboratory data has led to different classifications that take into account serum immunoglobulin levels, flow cytometry characteristics of CVID B lymphocytes, or B lymphocyte function [38]. Phenotyping of B cell subpopulations has confirmed the reduction of switched memory B cells (IgM-IgD-CD27+CD19+) in association with splenomegaly or granulomatous disease [38]. Changes in the proportion of other subgroups of B lymphocytes have also been described in CVID [39–41].

We have now studied a series of adult CVID patients, trying to correlate clinical findings with some characteristics of the peripheral blood T lymphocyte phenotype. Patients were divided in two groups: those who had evidence of AI disease or GD (Group I) and those who did not (Group II).

Because T_FH_ are important for the setup of a correct antibody response, we have focused on this helper T cell subpopulation in relation to the occurrence of immune dysregulation associated with GD or AI disease. Interestingly, we observed that the number of CXCR5+CD4+ T cells was significantly related to the number of CD19+ B lymphocytes, in Group I patients (with a similar tendency in N), suggesting that there may be a link in the regulation of the number of these lymphocytes.

Our results clearly show that the T_FH_ population was expanded in both groups of CVID patients, but CXCR5 and PD-1 coexpression was higher in Group I than in Group II and N. PD-1 is a potent inhibitory receptor important for T cell tolerance and it has been associated with CD8 T cell exhaustion during chronic viral infection [7, 28, 42]. A subgroup of T_FH_ defined by high PD-1 and low CCR7 expression has been demonstrated in patients with autoimmune conditions [32]. These cells seem to be the counterpart of active effector CD4 T cells within the T_FH_ compartment and contrast with central memory/resting T_FH_ having high CCR7 expression and lower PD-1 that are present in higher numbers in N. In our series, T_FH_ exhibiting high PD-1 and low CCR7 were also more prominent in Group I than in Group II and N. Nevertheless, it is interesting to note that T_FH_ from Group II patients without AI or GD had also lower CCR7 and higher PD-1 expression than N. Long term follow-up of these patients may give an insight into the relationship of T_FH_ and development of AI or GD in CVID.

Recently, the regulatory role of T_FH_ lymphocytes bearing the Treg transcription factor FoxP3 and CXCR5 (T_FH_ reg) in the generation of humoral immunity has been emphasized [43]. It was found that PD-1/PD-ligand 1 (PDL-1) interactions affected the outcome of T/B cooperation. PD-1 deficient mice had more T_FH_ reg than wild type mice and these PD-1 deficient T_FH_ reg had enhanced ability to suppress T_FH_ function [43]. Apparently, overexpression of FoxP3+ CD4+ T cells (either Treg or T_FH_ reg) was not the case in CVID, since neither Treg nor T_FH_ reg values differed from those of N in both CVID groups.

In addition to detailed studies on B lymphocyte phenotype and differentiation in CVID patients, the function of both CD4+ and CD8+ T lymphocytes has been thoroughly studied in CVID. A defect in the integration of activating signals derived from the TCR and costimulatory molecules in CD4 and CD8 T lymphocytes in CVID patients was demonstrated [44]. Furthermore, it was shown that the proliferative response of purified CD4 and CD8 T lymphocytes to recall antigens and superantigens was impaired due to a defect in the early phase of T cell receptor-mediated T cell activation [45]. Antigen presentation by CVID monocytes or B lymphocytes was unaffected in CVID [46]. Our studies have been focused on the action of a nonmitogenic dose of PHA on T_FH_ in culture. There are important differences in this experimental procedure and the ones reported before. We did not evaluate the proliferative response of PBMC and we did not work with purified cell populations (CD4 or CD8) as did the previous studies. In spite of their increased numbers, the function of T_FH_ could have been impaired in CVID. Our results demonstrate that T_FH_ cells (in the presence of the monocytes and B lymphocytes that coexist in PBMC) are able to upregulate the costimulatory molecules CD40L and ICOS and can respond in vitro by producing the cytokines involved in T-B cooperation to the same extent in Group I and Group II patients as in N. This does not imply that upon physiologic TCR stimulation this will be occurring in vivo. Intracellular synthesis of IL-10 and IL-21 (cytokine markers of T_FH_ cells) as well as that of IL-4 was similar to that of N in PHA-stimulated CVID CD4+CXCR5+ T lymphocytes. Notably, intracellular expression of IL-10, IL-21, and IL-4 was also higher than N in PHA-stimulated CXCR5-negative CD4+ T cells from CVID patients. In this regard, an important role of IL-21 (either from T_FH_ or from other cell sources) as a player in the differentiation of Th17 cells that are important in autoimmunity has been recently proposed [47]. It is possible that an IL-21-stimulated Th17 response could play a role in the generation of AI or EG in CVID patients. Because T_FH_ comprise a heterogeneous group of cells, increased circulating CXCR5+CD4+ T lymphocytes might not reflect their ability to cooperate with antibody production. T_h2_T_FH_ are required for adequate antibody synthesis, while T_h1_T_FH_ are not [24]. The fact that after stimulation with PHA CXCR5+CD4+ T cells from CVID patients produced IL-4 in addition to IL-21 and IL-10 indicates that lack of T_h2_T_FH_ was not the cause of the immunologic defect in this case.

High T_FH_ in circulation has also been reported in some autoimmune conditions [48] and this is interesting in relation to the increased occurrence of autoimmune phenomena in CVID. Indeed, in juvenile dermatomyositis (JDM), immune dysregulation leads to skewing of blood CXCR5+ T_h_ (T_FH_) subsets towards T_h2_ and T_h17_T_FH_ [24], while in JDM and other autoimmune conditions as systemic lupus erythematosus (SLE) or Sjogren's disease, increased levels of functional CXCR5+CD4+ T cells lead to hypergammaglobulinemia and autoantibody synthesis [49–51] in CVID hypogammaglobulinemia or selected immunoglobulin deficiency occurs, probably as a result of impaired B cell maturation [52]. On the other hand, repeated infections or continuous activation of the inflammatory response [53] could also determine the T_FH_ increase, since circulating T_FH_ with an active effector profile (high PD-1, low CCR7) were highest in patients with GD, AI, or both GD and AI manifestations.

## 5. Conclusions

Our results demonstrate that circulating T_FH_ are higher than normal in adult CVID patients. T_FH_ were able to respond to stimulation upregulating costimulatory molecules and providing the appropriate B helping cytokines. This suggests that intrinsic functional T_FH_ defects are not the primary cause of CVID. Moreover, increased levels of circulating T_FH_ highlight the relationship of CVID with other autoimmune diseases.

## Figures and Tables

**Table 1:** Clinical and epidemiological data of Group I patients.

Patient	Sex	Age (years)	Age at onset (years)	Age at Dx. (years)	Follow up D. (years)	GD	AI
1	F	65	31	41	24	Larynx, bowel, conjuntiva, skin	Hypothyroidism
2	M	36	1	13	23	LIP, LN	AHA
3	F	21	16	17	4	0	ITP, AHA
4	M	69	50	66	3	Lung, bowel	ITP
5	F	69	ND	66	3	LIP	0
6	F	36	ND	20	16	0	E N
7	M	30	ND	14	16	Lung, LN	ALOP
8	F	59	25	43	16	0	AHA, N

LN: lymph node; LIP: Lymphocytic interstitial pneumonia. LIP diagnoses were made by pathological examination in patients #1, #2 and #7. LIP in patient #4 was established by CAT scan. AHA: autoimmune hemolytic anemia; ALOP: alopecia aereata; ITP: immune thrombocytopenia; EN: erythema nodosum, N: autoimmune neutropenia.

**Table 2:** Expression of costimulatory molecules (CD40L and ICOS) in PHA+IL-2 stimulated PBMC from CVID patients and N.

	Costimulatory molecules expression in CD4+ lymphocytes (%, mean ± SE)			
	CD40L+	CXCR5+CD40L+	ICOS	CXCR5+ICOS+
Group I (*n* = 4)				
Medium	1.8 ± 2	1.7 ± 1.2	3.0 ± 0.2	2.2 ± 1.7
PHA+IL-2	11.0 ± 3.7	9.4 ± 3.5	24.4 ± 8.2	17.0 ± 5.3
Group II (*n* = 4)				
Medium	2.4 ± 2	1.9 ± 1.1	4.3 ± 1.8	3.0 ± 1.7
PHA+IL-2	11.5 ± 4.1	10.3 ± 4.2	22.8 ± 9.0	15.6 ± 6.5
Normals (*n* = 7)				
Medium	2.3 ± 1.5	2.0 ± 1.3	2.8 ± 3.2	1.8 ± 0.9
PHA+IL-2	7.3 ± 1.5	6.4 ± 1.5	16.3 ± 1.8	7.2 ± 1.1

PBMC were stimulated with 2.5 *μ*g/mL phytohemmagglutinin and 5 UI/mL interleukin-2 for 2 days and then were stained with anti-CD4, anti-CXCR5 and either anti-CD40L or anti-ICOS. The percentage of CD4+ cells that express CD40L, CXCR5 plus CD40L, ICOS and CXCR5 plus ICOS is consigned. No significant differences were observed between both CVID groups and Normals.

## Abbreviations

CVID: Common variable immune deficiency
T_FH_: T follicular helper cells
GD: Granulomatous disease
AI: Autoimmunity
ICOS: Inducible costimulator
T_FH_ reg: Regulatory T follicular helper cells.
